# Interferon-****α**** Enhances 5′-Deoxy-5-Fluorouridine-Induced Apoptosis by ERK-Dependant Upregulation of Thymidine Phosphorylase

**DOI:** 10.1155/2013/132793

**Published:** 2013-08-20

**Authors:** Yike Zhu, Ling Xu, Yibo Fan, Ce Li, Ye Zhang, Huachuan Zheng, Kezuo Hou, Xiujuan Qu, Yunpeng Liu

**Affiliations:** ^1^Department of Medical Oncology, The First Hospital of China Medical University, Shenyang, Liaoning 110001, China; ^2^Department of Biochemistry and Molecular Biology, College of Basic Medicine, China Medical University, Shenyang, Liaoning 110001, China

## Abstract

5-Florouracil (5-FU) is the basic agent used in the treatment of gastric cancer. Capecitabine, a prodrug of 5-FU, displays increased antitumor efficacy compared with 5-FU in the clinic. 5′-Deoxy-5-fluorouracil (5′-DFUR), the metabolite of capecitabine, is converted to 5-FU by the enzyme thymidine phosphorylase (TP), which is present at high concentrations in human tumors. In this study, we investigated the effect of interferon-**α** (IFN-**α**) on the sensitivity of gastric cancer cells to treatment with 5′-DFUR and its relationship with TP expression. Preincubation of gastric cancer cells with IFN-**α** enhanced 5′-DFUR-induced apoptosis via IFN-**α**-mediated upregulation of TP. The depletion of TP with small interfering RNA (siRNA) obviously inhibited IFN-**α**-induced upregulation of TP expression and thus prevented apoptosis induced by IFN-**α** and 5′-DFUR. Treatment with IFN-**α** and combined IFN-**α** and 5′-DFUR treatment were also associated with concomitant activation of ERK signaling. Treatment with the ERK inhibitor PD98059 or depletion of ERK with siRNA partially reversed IFN-**α**-induced upregulation of TP expression, thus partially preventing apoptosis induced by IFN-**α** and 5′-DFUR. Taken together, our study shows that IFN-**α** enhanced 5′-DFUR-induced apoptosis in gastric cancer cells by upregulation of TP expression, which is partially regulated by activation of ERK signaling.

## 1. Introduction

Gastric cancer is one of the most common malignant tumors worldwide, particularly in Eastern Asian countries such as China, Japan, and Korea [1]. Although 5-florouracil- (5-FU-) based combinational chemotherapy has improved survival for patients with advanced gastric cancer, the prognosis in cases of advanced disease remains poor [2]. A recent meta-analysis based on two large phase III REAL-2 and ML17032 trials indicated that treatment of advanced gastric cancer patients with capecitabine (an oral fluoropyrimidine carbamate) was superior to 5-FU in terms of overall survival [3]. 5′-Deoxy-5-fluorouridine ribose (5′-DFUR), the intermediate metabolite of capecitabine, is converted to 5-FU by thymidine phosphorylase (TP), an enzyme found at higher concentrations in tumors compared with normal tissues [4]. Higher activity of TP allows 5′-DFUR to be specifically targeted to the site of the cancer, leading to relatively high local concentrations of 5-FU in tumor cells [5, 6]. Thus, enhancing TP expression may represent an important strategy for increasing the antitumorigenic effect of 5′-DFUR.

Interferon-*α* (IFN-*α*) plays an essential role in antiviral responses and has been used clinically for the treatment of viral infections [7]. When administered alone, IFN-*α* can improve the immune function of the body and has been used for the treatment of myeloproliferative diseases in addition to solid tumors, such as renal cell carcinoma and melanoma [8–10]. In addition, chemotherapy with sequential IFN-*α* treatment prolonged survival in adult T-cell leukemia/lymphoma [11]. Colon cancer patients treated with both IFN-*α* and 5-FU showed a trend towards improved recurrence-free survival [12]. However, the efficacy of combination IFN-*α* and chemotherapy in gastric cancer has not been reported. Previous studies have shown that IFN-*α* enhances the antitumor effect of capecitabine on hepatocellular carcinoma in nude mice [13]. Moreover, IFN-*α* has been shown to increase the sensitivity of renal carcinoma cells to 5′-DFUR-induced apoptosis by enhanced TP expression [14]. However, whether the induction of TP mediated by IFN-*α* is a common phenomenon in other cancers remains unknown. Moreover, the mechanism by which IFN-*α* upregulates TP expression remains to be elucidated.

In the present study, we show that IFN-*α* sensitizes gastric cancer cells to 5′-DFUR-induced apoptosis by upregulation of TP expression, which is partially regulated by activation of the extracellular-regulated protein kinase (ERK) pathway.

## 2. Methods

### 2.1. Reagents and Antibodies

Recombinant human IFN-*α* was purchased from Prospec. 5′-DFUR was provided by Nippon Roche Co., Ltd (Tokyo, Japan). 3-(4,5-Dimethylthiazol-2-yl)-2,5-diphenyl tetrazolium bromide (MTT), dimethyl sulfoxide (DMSO), and the specific ERK inhibitor PD98059 were from Sigma (St. Louis, MO, USA). Antibodies specific for TP (sc-47702, Lot no. B0309), ERK (sc-153, Lot no. C0410), p-ERK 1/2 (Thr 202/Tyr 204)-R (sc-16982-R, Lot no. I1312), and Actin-R (sc-1616-R, Lot no. G0612) were purchased from Santa Cruz Biotechnology (Santa Cruz, CA, USA). Antibodies specific for Akt (9272S, Lot no. 24) and p-Akt (Ser 473) (9271L, Lot no. 13) were from Cell Signaling Technology (Danvers, MA, USA).

### 2.2. Cell Culture

The human gastric cell lines SGC7901 and MGC803 were obtained from Academy of Military Medical Science (Beijing, China). SGC7901 and MGC803 cells were grown in RPMI 1640 (Rosewell Park Memorial Institute) medium containing 10% heat-inactivated fetal calf serum (FCS) in a 37°C humidified incubator with a mixture of 95% air and 5% CO_2_.

### 2.3. MTT Assay

The cells were seeded in 96-well plates and then exposed to IFN-*α* and/or 5′-DFUR. Thereafter, 25 *μ*L of MTT solution (5 mg/mL) was added to each well, and the cells were incubated for another 4 h at 37°C. Then, the cells were lysed in 200 *μ*L of DMSO, and the optical density (OD) was measured at 570 nm with a microplate reader (Model 550, Bio-Rad Laboratories, USA). IC_50_ values were calculated using the probit model. The inhibition rate of cell proliferation was calculated as follows: inhibition rate (%) = 1 − *A*_570_ (test)/*A*_570_ (control) × 100%.

### 2.4. Flow Cytometry Analysis

The cells were cultured in the presence of IFN-*α* and/or 5′-DFUR for the indicated times. Then, the cells were then harvested and fixed with ice-cold 70% ethyl alcohol at 4°C overnight. After centrifugation at 2000 ×g for 5 min, the cell pellet was washed with PBS and incubated with RNase A (20 *μ*g/mL) at 37°C for 30 min. Next the cells were incubated with PI (10 *μ*g/mL) for 30 min in the dark. Finally, the samples were evaluated by flow cytometry, and the data were analyzed with CellQuest software (Becton Dickinson, San Jose, CA, USA).

### 2.5. Western Blot Analysis

The cells were solubilized in 1% Triton lysis buffer on ice. Lysates were collected after centrifuging at 12,000 rpm for 20 min at 4°C. Protein levels were quantified using Lowry method. Cell lysate proteins were separated by polyacrylamide gel electrophoresis and electrophoretically transferred to nitrocellulose membrane (Immobilon-P, Millipore, Bedford, MA, USA). The membranes were blocked with 5% skim milk in TBST buffer, incubated with the indicated antibodies, and reacted with horseradish-peroxidase-conjugated secondary antibodies. The proteins were detected with enhanced chemiluminescence reagent (SuperSignal Western Pico Chemiluminescent Substrate; Pierce, USA) and visualized with the Electrophoresis Gel Imaging Analysis System (DNR Bio-Imaging Systems, Israel).

### 2.6. Small Interfering RNA Transfections

TP small interfering RNA (siRNA) was obtained from Shanghai GeneChem Co. Ltd (China). TP siRNA was synthesized: 5′-AUAGACUCCAGCUUAUCCA-3′ (sence) and 5′-UGGAUAAGCUGGAGUCUAU-3′ (antisence). ERK small interfering RNA (siRNA) was obtained from Shanghai GenePharma Co. Ltd (China). ERK siRNA was synthesized: 5′-GUGCUCUGCUUAUGAUAAU-3′ (sence) and 5′-AUUAUCAUAAGCAGAGCAC-3′ (antisence). Lipofectamine 2000 was diluted dropwise into RPMI 1640 and incubated at room temperature for 5 min. Then TP or ERK siRNA was added to the diluted lipofectamine 2000 and incubated for another 20 min. After 48 h of transient transfection, the cells were analyzed by Western blot for TP or ERK siRNA effect.

### 2.7. Statistical Analysis

Data were confirmed in three independent experiments and were expressed as the mean ± standard deviation (SD). The significance of the difference between the groups was assessed by the Student's two tailed *t*-test. The statistical analyses were performed using the SPSS software 18.0 (SPSS Inc., Chicago, IL, USA). *P < 0.05 was considered statistical significance.

## 3. Results

### 3.1. IFN-*α* Enhances 5′-DFUR-Induced Apoptosis in Gastric Cancer Cells

**Figure 1:**
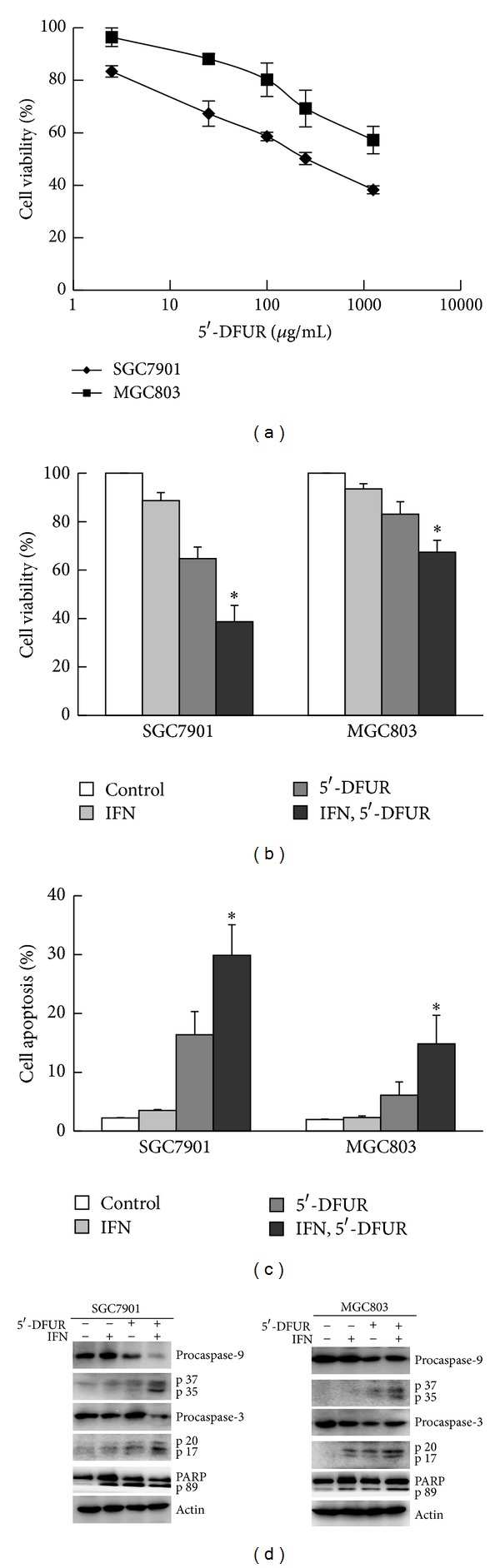
IFN-*α* enhanced 5′-DFUR-induced apoptosis in SGC7901 and MGC803 cells. (a) SGC7901 and MGC803 cells were treated with 2.5 *μ*g/mL, 25 *μ*g/mL, 100 *μ*g/mL, 250 *μ*g/mL, or 1250 *μ*g/mL 5′-DFUR for 48 h. The cell viability was examined by MTT assay. (b) SGC7901 and MGC803 cells were preincubated with 1000 IU/mL of IFN-*α* for 48 h, followed by treatment with 250 *μ*g/mL 5′-DFUR for 48 h. Quantitative representation of the cell viability. Data were means ± SD of three independent experiments. *Incubated with IFN-*α* and 5′-DFUR versus that with 5′-DFUR alone, *P* < 0.05. (c) SGC7901 and MGC803 cells were treated as described in (b). Cell apoptosis was quantified with flow cytometry. Data were means ± SD of three independent experiments. *Incubated with IFN-*α* and 5′-DFUR versus that with 5′-DFUR alone, *P* < 0.05. (d) SGC7901 and MGC803 cells were treated as described in (b). The expression of caspase-3, caspase-9, and PARP was analyzed by Western blot.

To investigate the effects of 5′-DFUR on the growth of gastric cancer cells, SGC7901 and MGC803 cells were treated with various doses of 5′-DFUR for 48 h. As shown in Figure 1(a), cell viability was inhibited by 5′-DFUR in a dose-dependent manner. The IC_50_ of 5′-DFUR was 273 ± 28 *μ*g/mL in SGC7901 cells, while the IC_50_ dose was not attained in MGC803 cells. To elucidate the effect of IFN-*α* on 5′-DFUR-induced inhibition of cell proliferation, SGC7901 and MGC803 cells were preincubated with IFN-*α* (1000 IU/mL) for 48 h, followed by treatment with 5′-DFUR (250 *μ*g/mL) for 48 h. Compared with treatment with 5′-DFUR alone, preincubation with IFN-*α* significantly enhanced the cytotoxicity of 5′-DFUR in both SGC7901 and MGC803 cells (*P* < 0.05, Figure 1(b)). While treatment of cells with IFN-*α* alone had no significant effect on apoptosis (~5% apoptosis), preincubation with IFN-*α* significantly enhanced 5′-DFUR-induced apoptosis in SGC7901 and MGC803 cells compared with 5′-DFUR alone (29.89 ± 5.18% versus 16.40 ± 3.93%, 14.83 ± 4.86% versus 6.10 ± 2.26%, resp., *P* < 0.05, Figure 1(c)). We also observed an increase in cleaved caspase-3, caspase-9, and PARP in cells treated with both IFN-*α* and 5′-DFUR, further confirming the induction of apoptosis (Figure 1(d)). These results demonstrate that IFN-*α* increases 5′-DFUR-induced apoptosis in gastric cancer cells.

### 3.2. IFN-*α* Upregulates TP Expression in Gastric Cancer Cells

**Figure 2:**
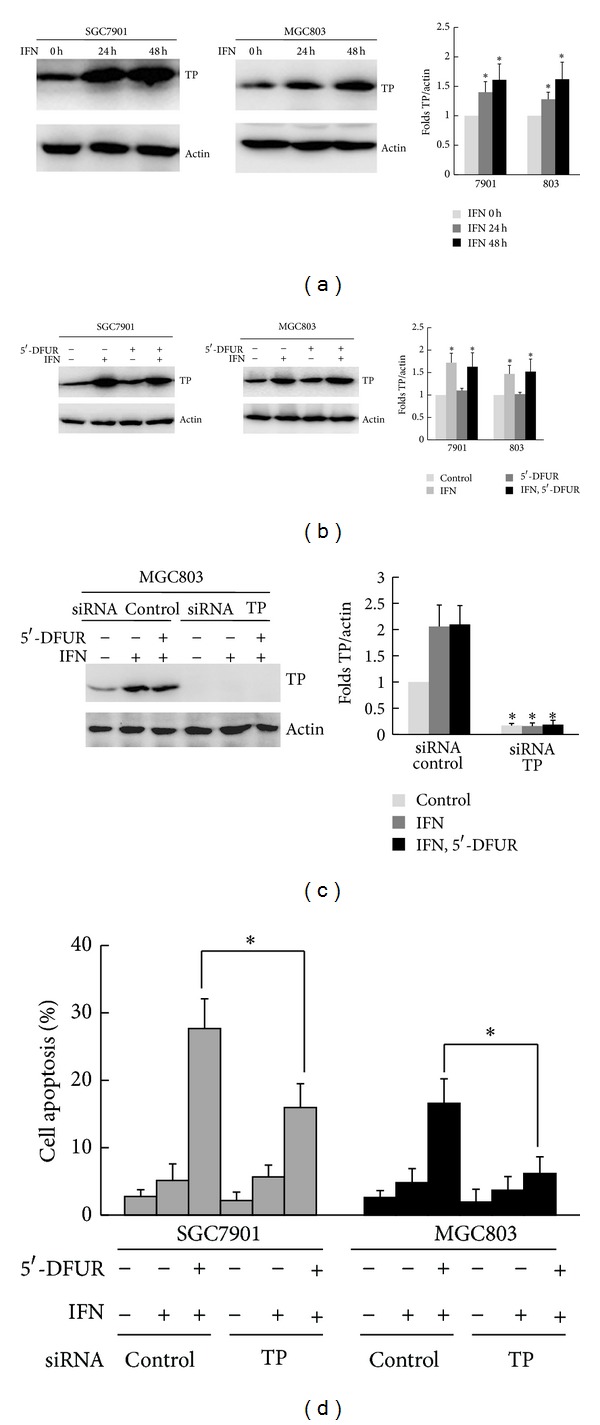
IFN-*α* upregulated the expression of TP in gastric cancer cells. (a) SGC7901 and MGC803 cells were treated with 1000 IU/mL of IFN-*α* for 24 h and 48 h. The expression of TP proteins was analyzed by Western blot. Data were means ± SD of three independent experiments. *Incubated with IFN-*α* for 24 h or 48 h versus that with IFN-*α* for 0 h, respectively, *P* < 0.05. (b) SGC7901 and MGC803 cells were preincubated with 1000 IU/mL of IFN-*α* for 48 h, followed by treatment with 250 *μ*g/mL of 5′-DFUR for 48 h. The expression of TP proteins was analyzed by Western blot. Data were means ± SD of three independent experiments. *Incubated with IFN-*α* alone or with IFN-*α* and 5′-DFUR versus that without both, respectively, *P* < 0.05. (c) MGC803 cells were transiently transfected with TP siRNA for 48 h, followed by 1000 IU/mL of IFN-*α* or 1000 IU/mL of IFN-*α* and 250 *μ*g/mL of 5′-DFUR. TP expression was analyzed by Western blot. Data were means ± SD of three independent experiments. *Incubated with IFN-*α* alone or with IFN-*α* and 5′-DFUR or without both in siRNA TP versus that in siRNA control, respectively, *P* < 0.05. (d) SGC7901 and MGC803 cells were transiently transfected with TP siRNA for 48 h, followed by 1000 IU/mL of IFN-*α* or 1000 IU/mL of IFN-*α* and 250 *μ*g/mL of 5′-DFUR. Cell apoptosis was quantified by flow cytometry. Data were means ± SD of three independent experiments. *Incubated with IFN-*α* and 5′-DFUR in siRNA TP versus that in siRNA control, *P* < 0.05.

To gain insight into the molecular mechanisms linking increased apoptosis to pretreatment with IFN-*α*, we examined the effect of IFN-*α* on the expression of TP in SGC7901 and MGC803 cells. As shown in Figure 2(a), exposure of SGC7901 and MGC803 cells to IFN-*α* led to induction of TP expression after 24 h. While exposure to 5′-DFUR alone did not significantly alter TP expression, the combined treatment with IFN-*α* and 5′-DFUR led to an increase in TP levels, similar to that induced with IFN-*α* alone (Figure 2(b)). To investigate whether IFN-*α*-induced TP upregulation is responsible for the effect of IFN-*α* and 5′-DFUR, we transiently transfected siRNA plasmids targeting TP into SGC7901 and MGC803 cells. Compared with siRNA controls, the depletion of TP with siRNA obviously inhibited IFN-*α*-induced upregulation of TP expression in MGC803 cells (Figure 2(c)). The similar results were also observed in SGC7901 cells (data not shown). As a single agent, TP siRNA had no significant effect on apoptosis. Compared with siRNA controls, the depletion of TP prevented apoptosis induced by IFN-*α* and 5′-DFUR in SGC7901 and MGC803 cells (27.68 ± 4.39% versus 15.96 ± 3.53%, 16.57 ± 3.65% versus 6.16 ± 2.48%, resp., *P* < 0.05, Figure 2(d)). These results indicate that IFN-*α* likely enhances 5′-DFUR-induced apoptosis via upregulation of TP expression.

### 3.3. IFN-*α* Induces the Activation of ERK in Gastric Cancer Cells

**Figure 3:**
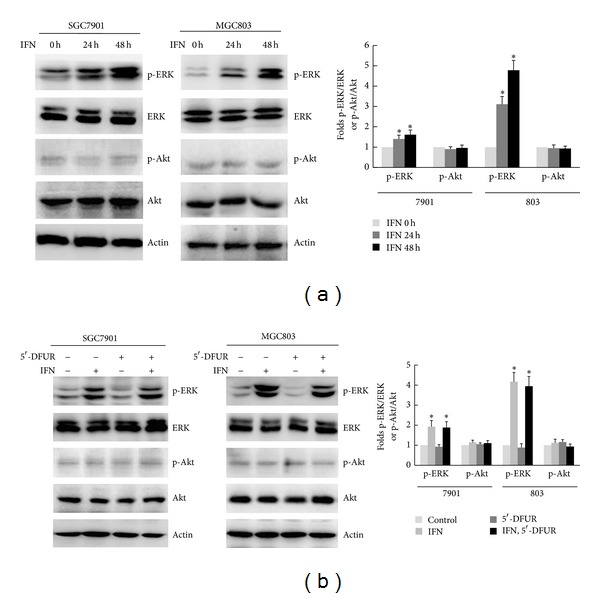
IFN-*α* induced ERK activation in gastric cancer cells. (a) SGC7901 and MGC803 cells were treated with 1000 IU/mL of IFN-*α* for 24 h and 48 h. The phosphorylation of ERK and Akt was analyzed by Western blot. Data were means ± SD of three independent experiments. *Incubated with IFN-*α* for 24 h or 48 h versus that with IFN-*α* for 0 h, respectively, *P* < 0.05. (b) SGC7901 and MGC803 cells were preincubated with 1000 IU/mL of IFN-*α* for 48 h, followed by treatment with 250 *μ*g/mL of 5′-DFUR for 48 h. The phosphorylation of ERK and Akt was analyzed by Western blot. Data were means ± SD of three independent experiments. *Incubated with IFN-*α* alone or with IFN-*α* and 5′-DFUR versus that without both, respectively, *P* < 0.05.

Recent studies have shown that the ERK pathway is linked to the expression of TP in nasopharyngeal carcinoma cells [15]. Based on this, we examined the effect of IFN-*α* on the activation of ERK signaling in gastric cancer cells. As shown in Figure 3(a), treatment of SGC7901 and MGC803 cells with IFN-*α* led to an increase in phosphorylated ERK (p-ERK) in a time-dependent manner. In contrast, we did not observe obvious change in the levels of phosphorylated Akt (p-Akt) following IFN-*α* treatment. Treatment of cells with 5′-DFUR alone did not significantly alter ERK activation; however, the combined treatment with IFN-*α* and 5′-DFUR led to activation of ERK, similar to that observed with IFN-*α* alone (Figure 3(b)). These data indicate that ERK activation may be involved in apoptosis induced by combined IFN-*α*- and 5′-DFUR treatment.

### 3.4. IFN-*α* Upregulates the Expression of TP Partially by Promoting ERK Activation in Gastric Cancer Cells

**Figure 4:**
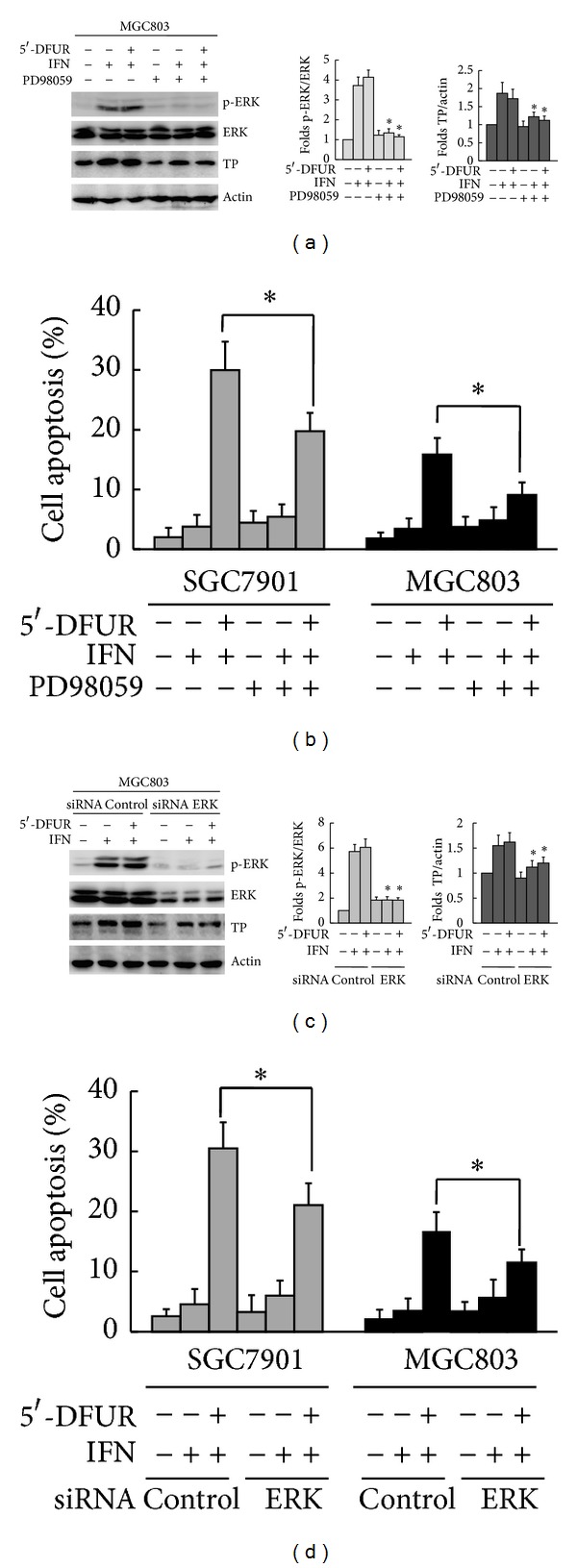
IFN-*α* upregulated the expression of TP partially by promoting ERK activation in gastric cancer cells. (a) MGC803 cells were preincubated with 20 *μ*mol/L ERK inhibitor PD98059 for 1 h followed by treatment with 1000 IU/mL of IFN-*α* or the combination of 1000 IU/mL IFN-*α* and 250 *μ*g/mL 5′-DFUR. Western blot analysis of the activated levels of ERK and the expression of TP. Data were means ± SD of three independent experiments. *Pretreated with PD98059 before IFN-*α* alone or IFN-*α*+5′-DFUR versus that untreated with PD98059 before IFN-*α* alone or IFN-*α* +  5′-DFUR, respectively, *P* < 0.05. (b) SGC7901 and MGC803 cells were treated as described in (a). Cell apoptosis was quantified with flow cytometry. Data were means ± SD of three independent experiments. *Pretreated with PD98059 before IFN-*α* and 5′-DFUR versus untreated with PD98059 before IFN-*α* and 5′-DFUR, *P* < 0.05. (c) MGC803 cells were transiently transfected with ERK siRNA for 48 h, followed by 1000 IU/mL of IFN-*α* or 1000 IU/mL of IFN-*α* and 250 *μ*g/mL of 5′-DFUR. The expression of proteins was analyzed by Western blot. Data were means ± SD of three independent experiments. *Incubated with IFN-*α* alone or with IFN-*α* and 5′-DFUR in siRNA ERK versus that in siRNA control, respectively, *P* < 0.05. (d) SGC7901 and MGC803 cells were transiently transfected with ERK siRNA for 48 h, followed by 1000 IU/mL of IFN-*α* or 1000 IU/mL of IFN-*α* and 250 *μ*g/mL of 5′-DFUR. Cell apoptosis was quantified by flow cytometry. Data were means ± SD of three independent experiments. *Incubated with IFN-*α* and 5′-DFUR in siRNA ERK versus that in siRNA control, *P* < 0.05.

To investigate whether IFN-*α*-induced ERK activation is responsible for the upregulation of TP expression, SGC7901 and MGC803 cells were exposed to IFN-*α* and 5′-DFUR in the presence or absence of ERK inhibitor PD98059. Preincubation with PD98059 for 1 h partially inhibited the induction of phosphorylated ERK mediated by treatment with IFN-*α* or IFN-*α*/5′-DFUR. Preincubation with PD98059 partially reversed the upregulation of TP induced by IFN-*α* and IFN-*α*+5′-DFUR in MGC803 cells compared with nontreated controls (Figure 4(a)). The similar results were also observed in SGC7901 cells (data not shown). While treatment with PD98059 alone did not obviously influence apoptosis induced by IFN-*α*, PD98059 partially inhibited apoptosis induced by IFN-*α* and 5′-DFUR in SGC7901 and MGC803 cells (29.98 ± 4.76% versus 19.73 ± 3.08%, 15.85 ± 2.75% versus 9.08 ± 2.12%, resp., *P* < 0.05, Figure 4(b)). To further determine the involvement of ERK activation in upregulation of TP expression, we transiently transfected siRNA plasmids targeting ERK into SGC7901 and MGC803 cells. Compared with siRNA controls, the depletion of ERK with siRNA partially inhibited IFN-*α*-induced upregulation of TP expression in MGC803 cells (Figure 4(c)). The similar results were also observed in SGC7901 cells (data not shown). As a single agent, ERK siRNA had no significant effect on apoptosis. Compared with siRNA controls, the depletion of ERK partially prevented apoptosis induced by IFN-*α* and 5′-DFUR in SGC7901 and MGC803 cells (30.50 ± 4.34% versus 21.07 ± 3.62%, 16.73 ± 3.15% versus 11.49 ± 2.18%, resp., *P* < 0.05, Figure 4(d)).These results indicate that IFN-*α* upregulates TP expression in part by activating ERK signaling and thus enhances 5′-DFUR-induced apoptosis.

## 4. Discussion

5′-DFUR is a precursor of 5-FU and is converted to 5-FU by TP. In the present study, we demonstrate that 5′-DFUR inhibits the proliferation of SGC7901 and MGC803 gastric cancer cells, at high concentrations. However, to enhance the sensitivity of gastric cancer cells to 5′-DFUR treatment, it is also necessary to increase the levels of TP. A recent study showed that TP expression may be clinically useful in predicting and improving the outcome of patients with head and neck squamous cell carcinoma treated with 5′-DFUR or capecitabine [16]. *In vitro* experiments also suggested that nasopharyngeal carcinoma tumors with high TP expression were sensitive to 5′-DFUR [17, 18]. IFN-*α* could improve the immune function of the body and has been used for the treatment of renal cell carcinoma, melanoma, and T-cell lymphoma. Continuous contact with PEG-IFN-*α*2b induces strong antitumor effects in human liver cancer cells *in vitro* and *in vivo* [19]. Makower et al. reported that interferon induced TP expression in peripheral blood mononuclear cells from tumor patients [20]. In addition, IFN enhances the cytotoxicity of 5′-DFUR in bladder cancer cells [21]. So, we analyzed the expression of TP in gastric cancer cells treated with IFN-*α* and 5′-DFUR. In the present study, we found that preincubation with IFN-*α* significantly enhanced the cytotoxicity of 5′-DFUR in SGC7901 and MGC803 gastric cancer cells compared with treatment with 5′-DFUR alone. Further experiments showed that IFN-*α* increased the expression of TP, and, similarly, combined treatment with IFN-*α* and 5′-DFUR led to induction of TP. Moreover, the depletion of TP with siRNA inhibited IFN-*α*-induced upregulation of TP expression and thus prevented apoptosis induced by IFN-*α* and 5′-DFUR. Thus, TP likely plays an important role in the enhancement of 5′-DFUR-induced apoptosis by IFN-*α* in gastric cancer cells.

Previous studies have shown that TP may be regulated by different signaling pathways, including protein kinase Cdelta, JNK, and ERK [22, 23]. While ERK activation is predominantly associated with survival and proliferation, ERK signaling has also been shown to play a role in apoptosis in some systems. Recently, we showed that VP-16 or Ara-c induces apoptosis in rat basophilic leukemia cells by enhancing MEK/ERK activation [24]. In addition, ERK is critical for apoptosis-associated mitochondrial events and apoptotic cell death induced by IFN-*α* in multiple myeloma cell lines [25]. In the present study, we show that IFN-*α* alone or in combination with 5′-DFUR upregulates ERK phosphorylation. Treatment with the ERK inhibitor PD98059 or ERK siRNA partially prevented IFN-*α*-induced phosphorylation of ERK and the upregulation of TP expression and thus partially inhibited IFN-*α* and 5′-DFUR-induced apoptosis. Our results indicate that ERK activation is one of upstream pathways of IFN-*α*-induced TP upregulation. So, ERK activation acts as an intermediary signaling molecule in the induction of apoptosis by IFN-*α* and 5′-DFUR.

## 5. Conclusions

Our data indicate that IFN-*α* is a potent sensitizer of 5′-DFUR-induced apoptosis in gastric cancer cells. This effect is mediated by induction of TP expression partially via ERK activation. These results provide important insights into the mechanisms underlying the effects of IFN-*α* and 5′-DFUR combination therapy in gastric cancer and may facilitate the design of new drug combinations.
